# Reliability of wearable sensors-based parameters for the assessment of knee stability

**DOI:** 10.1371/journal.pone.0274817

**Published:** 2022-09-22

**Authors:** Andrea Baldazzi, Luca Molinaro, Juri Taborri, Fabrizio Margheritini, Stefano Rossi, Elena Bergamini

**Affiliations:** 1 Department of Movement, Human and Health Sciences, Interuniversity Centre of Bioengineering of the Human Neuromusculoskeletal System, University of Rome “Foro Italico”, Roma, Italy; 2 Motustech–Sport & Health Technology, Rome, Italy; 3 Department of Economics, Engineering, Society and Business Organization (DEIM), University of Tuscia, Viterbo, Italy; Universita degli Studi di Pisa, ITALY

## Abstract

Anterior cruciate ligament (ACL) rupture represents one of the most recurrent knee injuries in soccer players. To allow a safe return to sport after ACL reconstruction, standardised and reliable procedures/criteria are needed. In this context, wearable sensors are gaining momentum as they allow obtaining objective information during sport-specific and in-the-field tasks. This paper aims at proposing a sensor-based protocol for the assessment of knee stability and at quantifying its reliability. Seventeen soccer players performed a single leg squat and a cross over hop test. Each participant was equipped with two magnetic-inertial measurement units located on the tibia and foot. Parameters related to the knee stability were obtained from linear acceleration and angular velocity signals. The intraclass correlation coefficient (ICC) and minimum detectable change (MDC) were calculated to evaluate each parameter reliability. The ICC ranged from 0.29 to 0.84 according to the considered parameter. Specifically, angular velocity-based parameters proved to be more reliable than acceleration-based counterparts, particularly in the cross over hop test (average ICC values of 0.46 and 0.63 for acceleration- and angular velocity-based parameters, respectively). An exception was represented, in the single leg squat, by parameters extracted from the acceleration trajectory on the tibial transverse plane (0.60≤ICC≤0.76), which can be considered as promising candidates for ACL injury risk assessment. Overall, greater ICC values were found for the dominant limb, with respect to the non-dominant one (average ICC: 0.64 and 0.53, respectively). Interestingly, this between-limb difference in variability was not always mirrored by LSI results. MDC values provide useful information in the perspective of applying the proposed protocol on athletes with ACL reconstruction. Thus, The outcome of this study sets the basis for the definition of reliable and objective criteria for return to sport clearance after ACL injury.

## Introduction

With an increasing worldwide incidence, anterior cruciate ligament (ACL) ruptures represent the most recurrent and critical injury related to the knee joint [1,2], accounting for 20% of total knee injuries [3] and around 50% of knee ligament tears [4]. ACL rupture is mostly common during sports requiring cutting and pivoting movements, like soccer, where the number of cases per year ranges between 10 and 35 per 1000 hours of practice [5].

Following an ACL rupture and surgical reconstruction, the athlete is involved in a long rehabilitation process, aiming at returning to unrestricted sports practice (RTS) in the safest possible way. However, a lack of consensus exists about the protocol and criteria/thresholds that must be used to determine the physical readiness to return to the preinjury activity level [6,7], leading to a very high probability of an ACL re-rupture. It has been found, in fact, that an ACL re-rupture is about 29.5% within two years after RTS, with 20.5% and 9.0% referring to contralateral and ipsilateral injury, respectively [8].

Traditionally, both laboratory- and functional/field-based assessments are performed for RTS clearance. Among the former, muscle force symmetry during isometric or isokinetic contractions is often considered [7,9]. However, these controlled contractions do not reflect typical isotonic contractions occurring during most sport-specific tasks. Conversely, functional and field-based tests allow for the reproduction of more realistic movements, like hop and agility tests based on change-of-direction movements [10–12]. Nevertheless, these tests are generally oriented to analyse the overall outcome rather than the quality of the execution from a biomechanical point of view. Specifically, they focus on the symmetry between injured and non-injured lower limb in terms of time to perform a test or jumping/hopping distance [11]. No information, however, are provided about the knee stability and, in turn, about the actual quality and safety of the movement itself [13,14]. This highlights the need to define new protocols and informative indices for the definition of RTS clearance after ACL reconstruction (ACLR). These protocols/indices must consider realistic sport-related movements and must provide quantitative and reliable information about subtle movements and knee stability.

In this perspective, wearable magneto-inertial measurement units (MIMUs) may represent a good compromise between laboratory- and field-based tests. MIMUs, which typically include 3D accelerometers, gyroscopes and magnetometers, may provide quantitative information during sport-specific, multi-joint and high intensity-based tasks. The application of MIMUs is extensive both in clinical movement analysis [15–18] and in sport performance contexts [19,20]. More recently, these sensors have also been applied for the investigation of risk factors associated to ACL injury. Either single- or multi-sensor approaches were adopted in the literature and different motor tasks were considered, from single leg squat [21–25], to drop landing/jumping or hopping [26–31] or side cutting manoeuvres [32–34]. Several parameters were also proposed, according to the adopted experimental protocol and to the final aim of the study: from knee joint kinematics and kinetics [31,34–36] to linear/angular velocity of both leg and thigh [25–27]. Few studies, though, focused on the assessment of the knee stability [23,24]. Furthermore, the reliability of the proposed parameters was rarely taken into account, undermining the effective applicability and usability of the proposed procedures. In addition, the existing literature mainly focused on single-task protocols and it did not consider both closed chain and hopping tests within the same protocol. Last but not least, the sampling frequency and full-range scale of the sensors used in most published studies is barely adequate for the analysis of impact and high jerked movements like jump landings.

The purpose of the present study is thus to propose a new wearable sensor-based protocol for knee stability assessment and to quantify the reliability of the proposed parameters. To this aim, two tests involving single-leg closed-chain movements typical of open skill sports were selected, i.e., the single leg squat and the crossover hop test. Both tests were performed by healthy soccer players wearing two MIMUs on the tibia and foot segments. We hypothesized that reliable parameters related to knee stability can be extracted from MIMU data during the selected motor tasks and that information about the reliability of those parameters will contribute to the definition of an effective assessment protocol for RTS clearance of athletes with ACLR.

## Materials and methods

### Participants

Seventeen healthy male soccer players (age: 21.5 ± 3.2 years (mean ± SD); stature: 1.84 ± 0.05 m; body mass: 74 ± 7 kg) participated in the present observational study. This sample size complies with an a priori power analysis performed with the following input parameters: minimum expected reliability = 0.3; expected reliability = 0.7; significance level = 0.05; power = 0.8, number of repetitions = 3 and resulting in 16 subjects [37]. Inclusion criteria were: (*i*) no history of significant lower limb injuries involving surgery or causing ongoing disability, (*ii*) 18 to 40 years of age, and (*iii*) Tegner Activity Score ≥ 6 [38]. The dominant lower limb was identified for each participant as the limb that was used to kick a ball twice [39]. All participants were right limb dominant, except one. Before starting data acquisition, a written consent was obtained from each participant. The study was approved by the University of Rome “Foro Italico” committee for research (CAR 19/2020).

### Instruments

**Fig 1:**
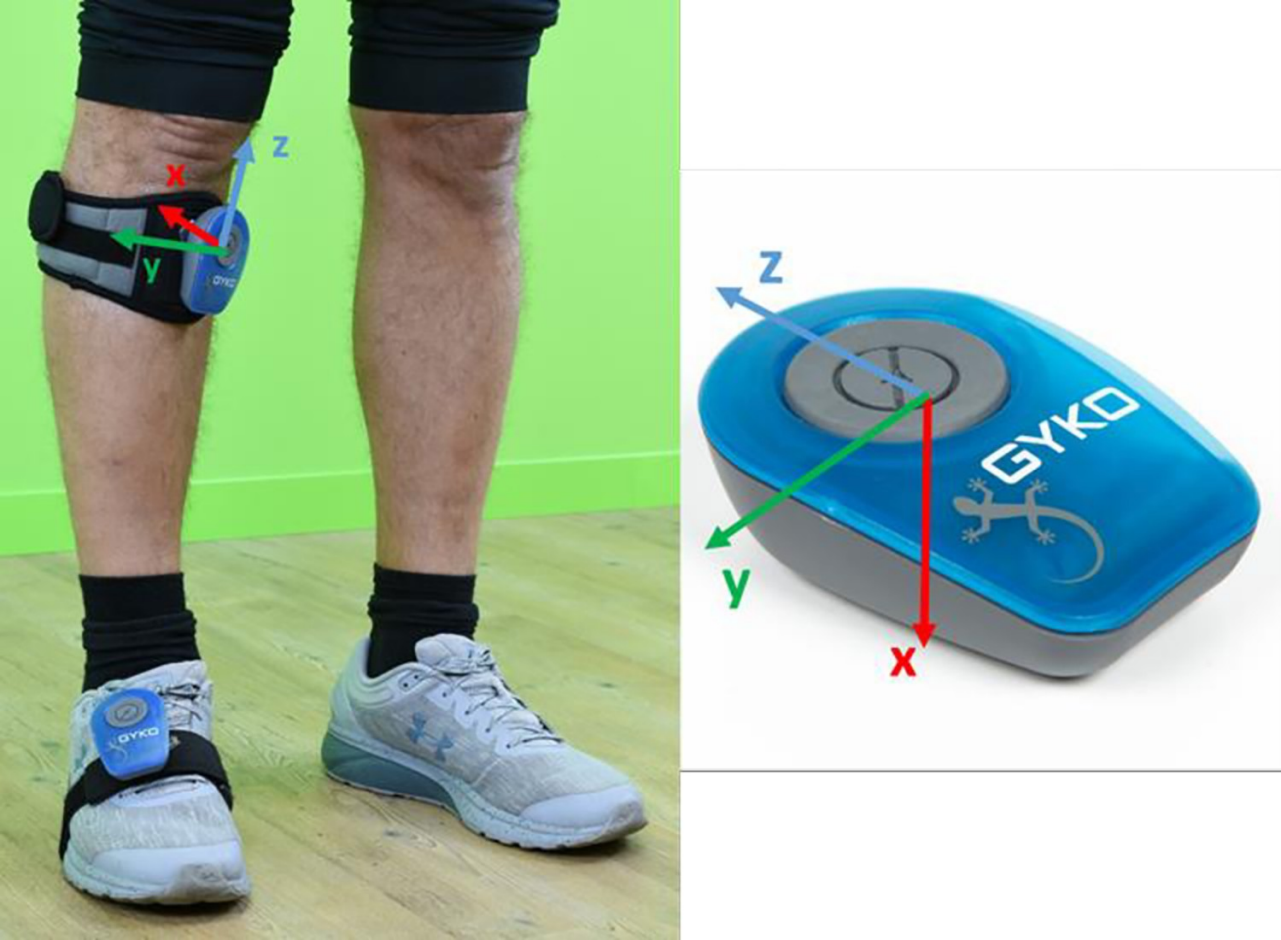
MIMU placement: ad hoc straps and tape were used to fix MIMUs on the foot and tibia.

Two MIMUs (Gyko, Microgate S.r.l., Italy) were attached on the foot dorsum and on the medial upper portion of the tibial crest of each participant using *ad hoc* straps (Fig 1), to limit their oscillations relative to the underlying segment [40].

MIMUs were positioned by the same operator and paying attention to align one of the MIMU axes (the x-axes) with the longitudinal axis of the relevant segment. Each MIMU includes 3D gyroscopes, linear accelerometers, and magnetic sensors (± 2000°/s, ± 16 g, and ± 4800 μT of full-range scale, respectively), measuring 3D angular velocity, linear acceleration, and magnetic field vector at a sampling frequency of 500 Hz. Considering the full-range scale and the sampling frequency, these MIMUs proved to be particularly suitable for the assessment of high-intensity sports movements [41,42]. Data acquired by the MIMUs were transmitted via Bluetooth to a laptop. The two MIMU data streams were electronically synchronized.

### Experimental protocol

Tests were conducted in the Laboratory of Bioengineering and Neuromechanics of Movement of the University of Rome “Foro Italico”. Before starting data acquisition, a standardized 5-minute warm up on cycle-ergometer was completed by each participant. Participants were first asked to perform two preliminary tests aimed at characterizing the population in terms of knee joint range of motion (RoM) and force exertion. For the former, the flexion-extension RoM of the knee joint was measured using a goniometer, with the subjects lying in a supine position, both for flexion and extension. Specifically, maximal knee joint extension was measured by asking the participants to lie in a supine position with their heel placed on a pillow, thus allowing for knee hyperextension, whereas maximal knee flexion was measured by asking participants to flex their knee in order to place the heel as close as possible to their gluteus [43]. For the force exertion, three countermovement jumps (CMJs) were performed by each participant on two force platforms (0.6 x 0.6 m each, AMTI, USA, sampling frequency: 1000 Hz). During the tests, participants started from an upright position, with each foot on a force platform and with their hands fixed on the hips [44]. They were asked to perform a maximal CMJ three times, with a pause of at least 20 s between two consecutive jumps. Parameters related to the vertical component of the ground reaction force (GRF) were then extracted for both limbs, as detailed in the Data processing section.

After the preliminary tests, each participant performed two functional tests: the Single Leg Squat (SLS) and the Crossover Hop Test (CHT). These two tests were selected since they include single-leg closed-chain movements, which are typical of the open skill sports. In addition, they are commonly proposed in the literature to assess readiness to RTS after an ACL injury and reconstruction [45,46]. A brief description of each test is reported below:

- *Single Leg Squat Test (SLS)* [45]. Starting from a standing bipodal position, participants were required to switch to a single leg support with the other leg lifted off the ground in front of the body. Participants were then asked to perform a squat. The arms were extended forward, and participants had to squat as much as they can maintaining balance and, successively, to get up to the initial bipodal position with knee fully extended.
- *Crossover Hop Test (CHT)* [46]. Participants were asked to hop forwards three times on a single leg while alternately crossing over a straight line marked on the floor, trying to cover the greatest distance as possible. They started from a bipodal standing position, switch to a single leg support and they were then asked to keep their hands on the hips throughout the entire test. The total hopped distance, from the start line to the heel of the landing leg, was measured.

Each test was performed three times for each side (the one instrumented with the MIMUs), resulting in six trials for each participant (three with the dominant limb and three with the non-dominant one). The order of the tests and the limb side were randomized for each participant to control for any order effects associated with repeated testing.

### Data preprocessing

Data acquired by the force plates and by each MIMU were analyzed using *ad hoc* developed Matlab® (The MathWorks Inc., Natick, MA, USA) scripts and functions. For MIMU data, the static bias of the gyroscope signals was calculated at the beginning of each trial, when both MIMUs were still, and removed from the whole signal [47]. Proper calibration of accelerometer and magnetometer was also verified according to the indication provided in [47].

#### Preliminary test–CMJ

**Fig 2:**
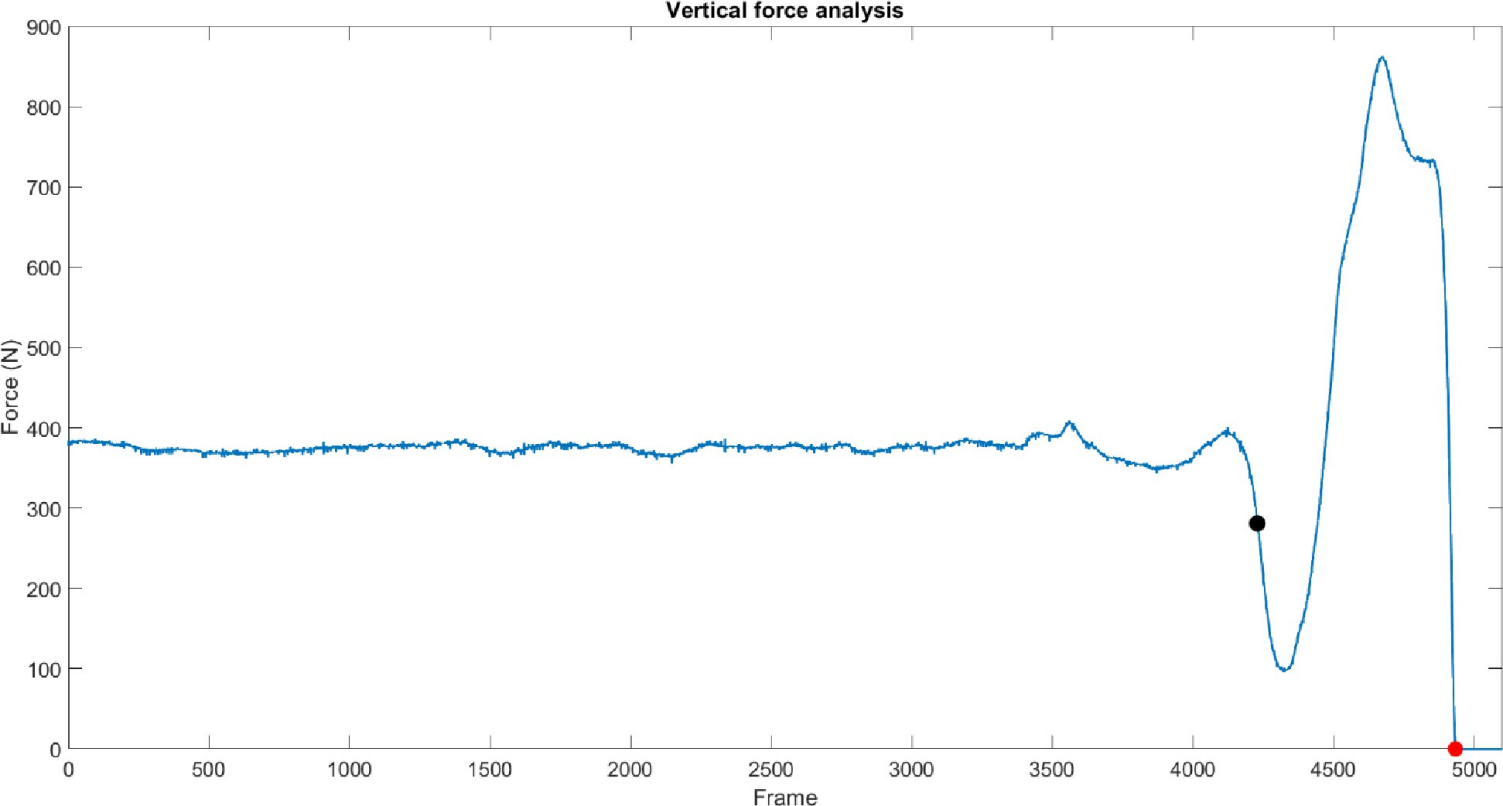
Analysis of the vertical force. Black square indicates the end of the unweighted phase whereas the red one the start of the time flight. As an example, the figure only reports the analysis of one of the two force platforms.

For each CMJ, the vertical component of the GRF measured by the force plates (GRFv) was considered for both legs. The time interval between the end of the unweighted phase and the start of the flight time was then identified for each trial and considered for the further analyses. The end of the unweighted phase was automatically identified as the instant in which the first inflection point of the curve occurred, whereas the start of the flight time was identified as the instant in which the vertical component was equal to zero, as in Fig 2. To obtain the net vertical force exchanged by each participant with the floor, participants’ weight was subtracted from the segmented data.

**Fig 3:**
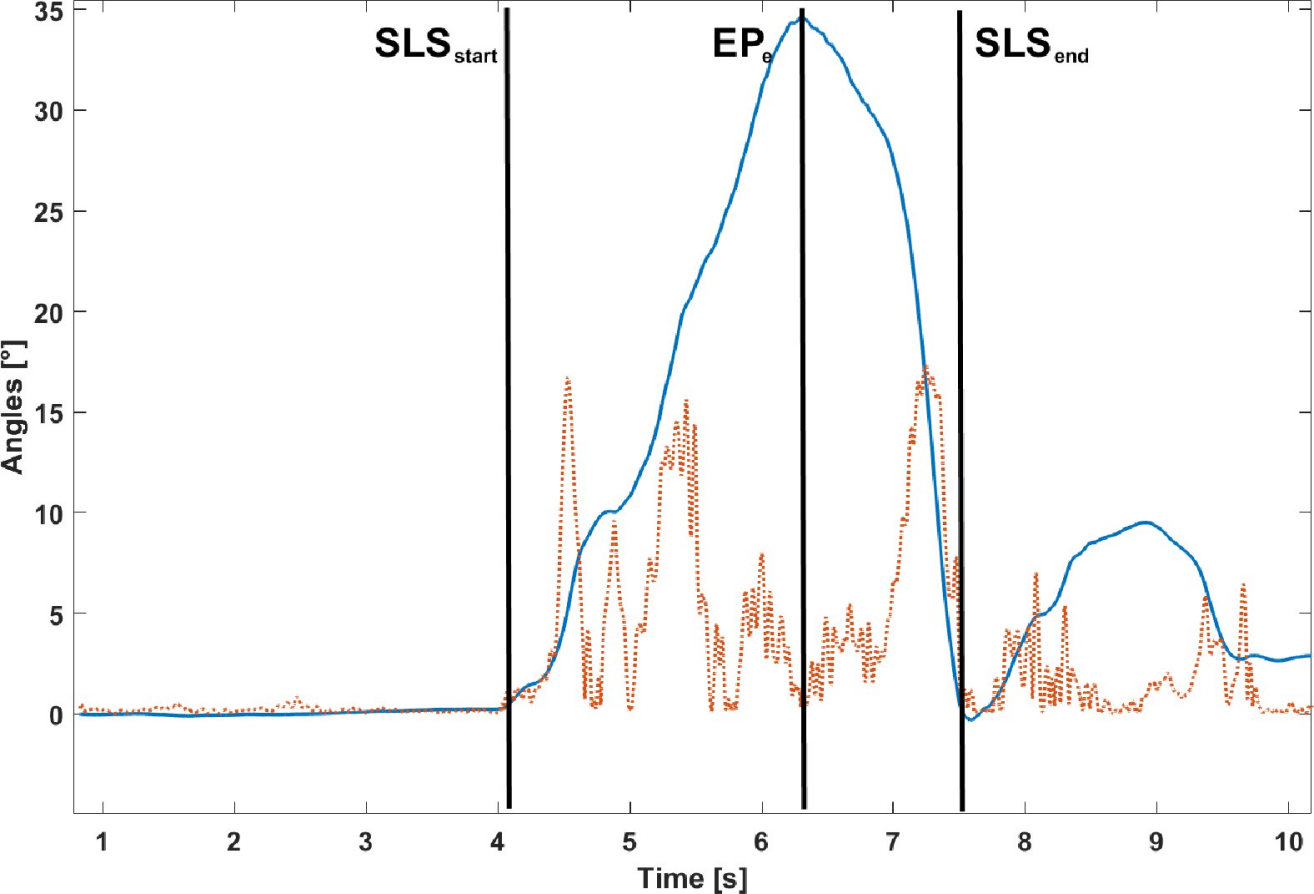
Angular displacement around the Y axis of the tibia MIMU (solid line) and its moving standard deviation of 10-sample window (dotted line). The three instants of time identified for SLS phase segmentation (SLS_start_, EP_e_, SLS_end_) are also depicted as vertical black lines.

**SLS.** For the SLS test, the angular displacement around the Y axis of the MIMU placed on the tibia (corresponding to the leg medio-lateral axis and approximately to the knee flexion axis) was obtained by means of a complementary filter (*k*_*1*_ = 2.33 and *k*_*2*_ = 7.44, where *k*_*1*_ and *k*_*2*_ are the coefficients that weight the confidence in the accelerometer and magnetometer measurements, respectively) [48]. Task segmentation entailed the identification of three instants of time: the beginning of the squat movement (SLS_start_), the end of the eccentric phase (EPe), and the end of the whole task (SLS_end_). To identify these instants, a moving standard deviation of 10-sample window (σ) was calculated on the above-mentioned angular displacement and multiplied by a factor of ten. The window size was selected based on a trial-and-error procedure, taking the sample frequency and the task dynamics into account. SLS_start_ was then defined as the instant in which σ > 2·σ_static_, where σ_static_ is the standard deviation of the angular displacement calculated in the first 1000 samples when the participant was still, before the task execution. EPe corresponded to the instant in which the maximum of the angular displacement curve occurred (Fig 2), whereas SLS_end_ was defined as the time instant when the angular displacement curve returned to its initial value, the same as in SLS_start_, i.e., the participant was back to his initial position (Fig 3).

#### CHT

**Fig 4:**
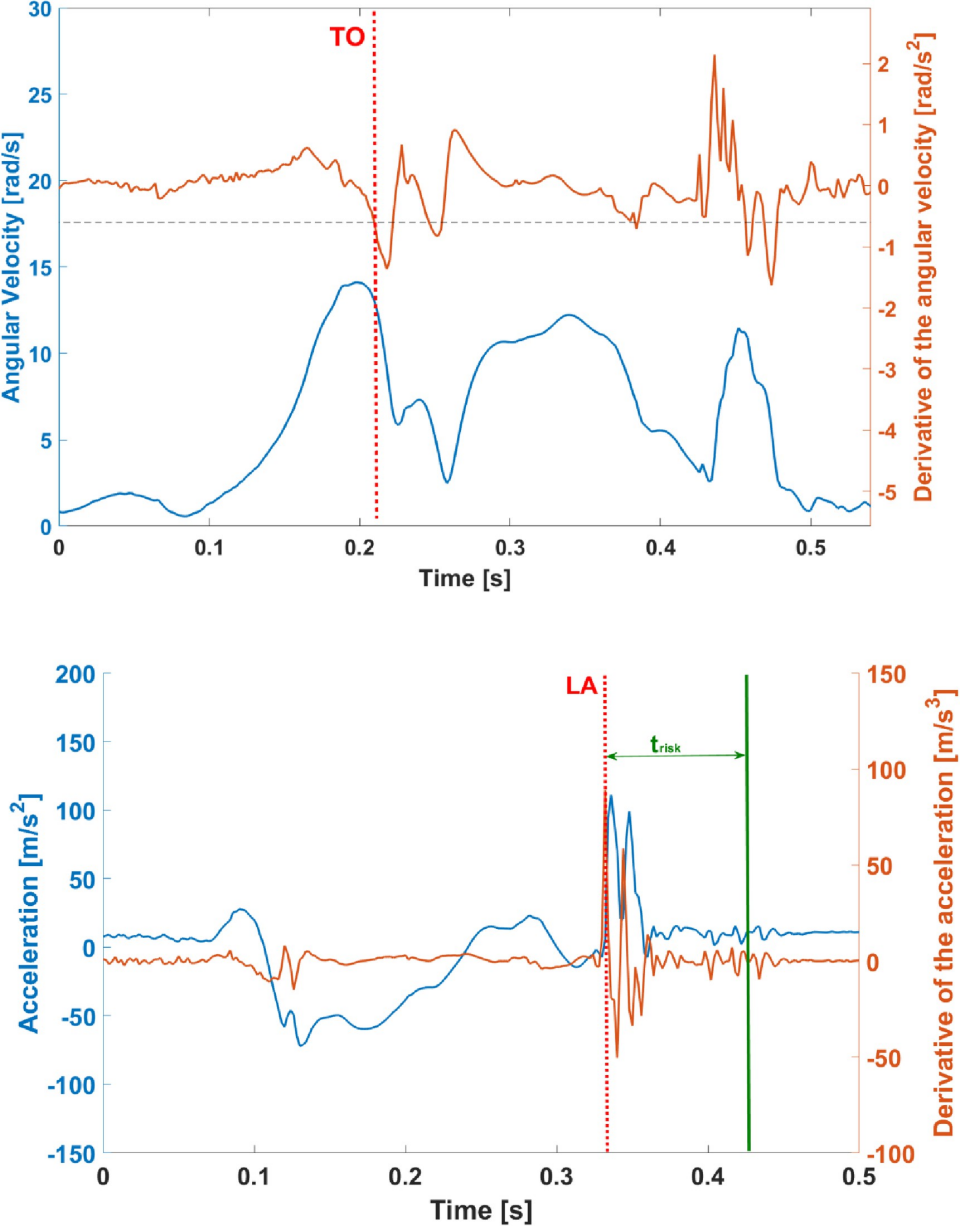
CHT phase segmentation: a) Top panel: Foot-based MIMU angular velocity magnitude (blue) and its derivative (orange) used to identify the take-off instant (TO, indicated with a red dashed line); b) Bottom panel: Foot-based MIMU vertical acceleration (blue) and derivative of the acceleration magnitude (orange) used to identify the landing instant (LA, indicated with a red dashed line). The 100 ms time window considered for the estimation of parameters related to the knee joint stability is also indicated (t_risk_ indicated as a green vertical line).

For the CHT, the acceleration measured by the MIMU located on the foot was expressed with respect to a vertically aligned inertial reference frame by means of the above-mentioned complementary filter [48]. The task was then segmented according to Ahmadian et al. [40] by identifying the take-off (TO) and landing (LA) time instants of each hop as follows: TO corresponded to the instant where the derivative of the angular velocity module of the foot sensor falls below a value of -0.6 rad/s^2^, whereas LA was identified as the first peak occurring in the first derivative of the acceleration norm having an amplitude greater than 7 m/s^3^ (Fig 4).

According to the existing literature, ACL injuries mostly occur in the first 100 ms after the contact of the foot with the ground [49–51]. Therefore, a time window of 100 ms (t_risk_) after LA was considered for the estimation of the parameters related to the knee joint stability (see Table 1).

**Table 1:** List of the MIMU-based parameters estimated for the Single Leg Squat test (SLS) and the Crossover Hop Test (CHT).

TYPE	PARAMETER	ACRONYM	MEAS. UNIT	DEFINITION	SLS	CHT
Temporal parameters	Total Test Duration	T_tot_	s	Time interval between the beginning and the end of the motor task. For the SLS, only the eccentric phase was considered.	**✓**	**✓**
	Flight Time	FT	s	LA_i_−TO_i-1_		**✓**
	Contact Time	CT	s	TO_i_−LA_i-1_		**✓**
Acceleration based stability parameters	Root Mean Square of the foot acceleration	RMSa_foot_	m/s^2^	${RMSa}_{foot}=\frac{1}{N}\sqrt{\sum_{i=1}^{N}a_{i\;foot}^{2}}$	**✓**	**✓**
	Root Mean Square of the leg acceleration	RMSa_leg_	m/s^2^	${RMSa}_{leg}=\frac{1}{N}\sqrt{\sum_{i=1}^{N}a_{i\;leg}^{2}}$	**✓**	**✓**
Angular velocity-based stability parameters	Root Mean Square of the foot angular velocity	RMSω_foot_	°/s	${RMS\omega }_{foot}=\frac{1}{N}\sqrt{\sum_{i=1}^{N}\omega_{i}^{2}}$	**✓**	**✓**
	Root Mean Square of the leg angular velocity	RMSω_leg_	°/s	${RMS\omega }_{leg}=\frac{1}{N}\sqrt{\sum_{i=1}^{N}\omega_{i}^{2}}$	**✓**	**✓**
	Peak of foot angular velocity	ωpeak_foot_	°/s	Peak of the angular velocity signal measured by the foot-mounted MIMU during T_risk_	**✓**	**✓**
	Peak of the leg angular velocity	ωpeak_leg_	°/s	Peak of the angular velocity signal measured by the leg-mounted MIMU during T_risk_	**✓**	**✓**
Parameters related to the tibia acceleration pattern on the YZ plane. [52–54]	Sway path of the leg acceleration	SP	m/s^2^	Total length of the acceleration trajectory on the plane described by the y-z acceleration components	**✓**	
	Sway Area	SA	m^4^/s^4^	Area of the 90% prediction ellipse which encloses approximately 90% of the points on the acceleration path SP	**✓**	
	Eccentricity of the 90% prediction ellipse used for the Sway Area calculation	SAecc	dimless	$SAecc=\sqrt{1-\frac{b^{2}}{a^{2}}}$ where *a* is the semi-major axis and *b* is the semi-minor axis of the 90% prediction ellipse used to calculate SA	**✓**	

### Parameter extraction

For the CMJs, two parameters were computed from the GRFv within the segmented time window: the maximum of the GRFv (F_max_) expressed in N, and the relative net impulse (AUC_F_), i.e. the net impulse normalised with respect the athletes’ body mass and thus expressed in m/s according to Kirby et al. [55].

For the SLS and CHT tests, temporal and knee-stability parameters were estimated. These parameters were based on both acceleration and angular velocity data measured by the tibia and foot MIMUs and they were selected based on the existing literature. More in detail, acceleration-based measures are known to be related to the knee stability and, specifically, the greater the acceleration the lower the stability of the joint [49,56]. On the other side, angular velocity peaks proved to be correlated with the external knee abduction moment, which in turn is considered a strong indicator of ACL injury risk [25,26,57]. In addition, acceleration-based parameters related to the knee excursion on the transverse plane were extracted [23,24,46]. The complete and detailed list of the estimated parameters is reported in Table 1. For the SLS, only the eccentric phase of the movement was considered, and all the estimated parameters were extracted within such a phase. For the CHT, all the acceleration- and angular velocity-based parameters were calculated during the t_risk_ interval, i.e., in a time window of 100 ms after jump landing (LA).

To investigate the presence of possible asymmetries between the dominant and non-dominant side, the Limb Symmetry Index (LSI) was calculated for all the extracted parameters as follows:

$$LSI=\frac{non\;dominant\;limb}{dominant\;limb}∙100$$ (1)

The LSI is commonly used to investigate asymmetries between the injured and non-injured limbs in the assessment of RTS readiness after ACL injury [9]. According to the existing literature, LSI values ranging from 85% to 115% are considered as indicators of physiological between-limb symmetry [58].

### Statistical analysis

To test the intra-participant reliability, the Intraclass Correlation Coefficient (ICC) was calculated for each parameter and each test, over the three trials, based on the absolute-agreement and the 2-way mixed-effects model [59]. The confidence interval of the ICC was also calculated considering a level of 95%. This analysis allowed for the quantification of the intra-subject variability in performing the same task in successive repetitions.

In order to quantify the minimum amount of change in the extracted parameters for ensuring that the change is not due to random and systematic errors, the Minimum Detectable Change (MDC) was also computed following the equation reported by Weir [60]:

$$MDC=SEM*1.96*\sqrt{2}$$ (2)

where 1.96 is the z-value for considering a confidence interval equal to 95% and SEM is the Standard Error of the Measurement computed as in the Eq 3:

$$SEM=SD\sqrt{1-ICC}$$ (3)

where SD is the standard deviation of the measurements. The MDC has been demonstrated a valid measure of reliability since it is independent from the population sample size and it is less affected by the between-subject variability with respect to the ICC.

For each task and each parameter, the median value over the three performed trials was extracted. The mean value and standard deviation across all the participants median scores were then calculated. For the CHT, only the second hop was considered for each trial, due to the high variability characterizing the take-off and landing technique related to the first and the third hops, respectively [61]. Statistical analysis was performed using the IBM SPSS Statistics software (v23, IBM Corp., USA), with the alpha level of significance set to 0.05 for all the tests.

## Results

### Preliminary tests

Knee joint RoM evaluation excluded functional limitations during knee joint flexion-extension. Maximal extension values were 2 ± 1° for both dominant and non-dominant limb, whereas maximal knee flexion was found to be 137 ± 1° and 138 ± 2° for the dominant and non-dominant limb, respectively. Results obtained during the CMJ tasks are reported in Table 2. Overall, a physiological symmetry between the dominant and non-dominant limb was found during a multi-articular closed-chain movement as the CMJ, both in terms of F_max_ and AUC_F_.

**Table 2:** Results of the two parameters estimated during the CMJ (mean ± standard deviation).

CMJ	DOM	NON-DOM	LSI [%]	ICC [CI 95%]	
				DOM	NON-DOM
F_max_ [N]	520.6 ± 73.6	482.0 ± 67.9	93 ± 10	0.88* [0.79–0.94]	0.87* [0.78–0.94]
AUC_F_ [m/s]	2.2 ± 0.2	2.1 ± 0.2	97 ± 8	0.93** [0.84–0.97]	0.92** [0.81–0.96]

The LSI and ICC values are also reported with the relative confidence interval at 95%: * p<0.05, ** p<0.01. DOM: Dominant limb, NON-DOM: Non dominant limb.

### SLS and CHT

Tables 3 and 4 report the results obtained from the analysis of the two functional tests (SLS and CHT), together with the ICC and MDC values for all the computed parameters. The values of the LSI for each estimated parameter are also reported. As expected, a physiological symmetry [58] was displayed by the participants for all the examined parameters and in both tests. For the CHT, the mean ± standard deviation values in terms of distance covered were 4.94 ± 0.58 m for the dominant limb and 4.91 ± 0.47 m for the non-dominant one, resulting in LSI of 99 ± 8%. The ICC values of the CHT covered distance were 0.71 and 0.83, for the non-dominant and dominant limb, respectively (p<0.01).

**Table 3:** Results of the parameters estimated during the SLS (mean ± standard deviation).

SLS	DOM	NON-DOM	LSI [%]	ICC [CI 95%]		MDC	
				DOM	NON-DOM	DOM	NON-DOM
T_tot_ [s]	2.2 ± 1.1	2.3 ± 0.8	109 ± 36	0.66* [0.39–0.84]	0.67** [0.37–0.87]	0.6	0.4
RMSa_foot_ [m/s^2^]	0.4 ± 0.4	0.2 ± 0.1	95 ± 45	0.74* [0.45–0.89]	0.29* [0.08–0.35]	0.2	0.1
RMSa_leg_ [m/s^2^]	0.6 ± 0.2	0.6 ± 0.3	102 ± 25	0.64* [0.37–0.82]	0.67* [0.41–0.80]	0.1	0.1
RMSω_foot_ [°/s]	11.4 ± 5.7	17.2 ± 5.6	112 ± 55	0.63* [0.39–0.82]	0.64* [0.38–0.83]	3.3	3.1
RMSω_leg_ [°/s]	28.6 ± 5.6	28.7 ± 5.7	103 ± 18	0.66** [0.41–0.82]	0.76* [0.50–0.89]	2.9	2.0
ωpeak_foot_ [°/s]	63.0 ± 40.1	51.6 ± 22.9	103 ± 53	0.68* [0.42–0.83]	0.50* [0.22–0.65]	19.5	18.6
ωpeak_leg_ [°/s]	74.5 ± 22.8	74.4 ± 28.7	103 ± 38	0.60* [0.32–0.80]	0.42 [0.15–0.60]	14.2	28.0
SP [m/s^2^]	192.3 ± 70.3	213.5 ± 87.3	113 ± 37	0.60** [0.36–0.79]	0.60* [0.35–0.75]	43.9	54.5
SA [m^2^/s^4^]	36.4 ± 22.7	37.7 ± 20.5	107 ± 36	0.76* [0.55–0.90]	0.65* [0.43–0.79]	8.1	11.0
SAecc [dimless]	0.8 ± 0.1	0.8 ± 0.1	97 ± 9	0.70** [0.42–0.87]	0.70* [0.45–0.85]	0.0	0.0

The LSI and ICC values are also reported with the relative confidence interval at 95%

* p<0.05

** p<0.01. DOM: Dominant limb, NON-DOM: Non dominant limb. MDC stands for Minimum Detectable Change.

**Table 4:** Results of the parameters estimated during the CHT (mean ± standard deviation).

CHT	DOM	NON-DOM	LSI [%]	ICC [CI 95%]		MDC	
				DOM	NON-DOM	DOM	DOM
T_tot_ [s]	1.6 ± 0.2	1.7 ± 0.2	103 ± 7	0.82** [0.61–0.89]	0.84* [0.63–0.94]	0.0	0.0
CT [s]	0.4 ± 0.1	0.4 ± 0.1	102 ± 15	0.74** [0.57–0.86]	0.77* [0.59–0.91]	0.0	0.0
FT [s]	0.3 ± 0.0	0.3 ± 0.0	103 ± 8	0.46 [0.29–0.59]	0.43* [0.20–0.58]	0.0	0.0
RMSa_foot_ [m/s^2^]	82.9 ± 9.9	76.4 ± 12.9	93 ± 13	0.45 [0.27–0.60]	0.42 [0.25–0.61]	9.0	12.6
RMSa_leg_ [m/s^2^]	67.8 ± 14.6	62.7 ± 21.9	88 ± 26	0.72* [0.59–0.81]	0.25 [0.02–0.44]	6.1	30.3
RMSω_foot_ [°/s]	509.9 ± 108.8	504.2 ± 108.9	96 ± 16	0.63* [0.49–0.77]	0.66* [0.48–0.89]	62.2	56.6
RMSω_leg_ [°/s]	429.7 ± 97.4	401.1 ± 97.6	91 ± 18	0.63* [0.40–0.81]	0.50* [0.32–0.75]	55.7	79.2
ωpeak_foot_ [°/s]	1294.9 ± 441.1	1168.8 ± 366.7	91 ± 23	0.70* [0.48–0.87]	0.65* [0.39–0.83]	199.7	197.0
ωpeak_leg_ [°/s]	830.8 ± 280.7	704.7 ± 275.0	84 ± 25	0.60** [0.39–0.84]	0.66* [0.40–0.81]	175.4	143.0

The LSI and ICC values are also reported with the relative confidence interval at 95%: * p<0.05, ** p<0.01. DOM: Dominant limb, NON-DOM: Non dominant limb. MDC stands for Minimum Detectable Change.

## Discussion and implications

The present study proposes a new instrumented protocol based on the use of wearable sensors for the assessment of knee stability and quantifies the reliability of the proposed parameters. In particular, temporal-, acceleration- and angular velocity-based parameters descriptive of the knee movement were proposed during two tests involving single-leg closed-chain movements typical of open skill sports: the SLS and CHT. For the SLS, root mean square values of foot- and leg-based angular velocity magnitude together with the acceleration pattern on the tibia transverse plane proved to be reliable parameters to quantify the knee stability. When the CHT is considered, temporal- and angular velocity- related parameters, like the total test duration, contact times and foot- and leg-based angular velocity magnitude peaks, displayed the highest reliability and they can thus be considered as promising candidates for ACL injury risk assessment.

Two preliminary tests were performed to characterize the population in terms of the knee joint RoM and force exertion (the latter, during CMJs). The results of these tests are in agreement with the existing literature [55,62], both in terms of mean values and reliability [63], and symmetry values proved to be within physiological ranges [58].

For the SLS test, the eccentric phase (from standing position to maximum knee flexion) was considered, since eccentric activation of the quadriceps is proven to increase stresses on ACL in sports practice [64]. The values obtained for the acceleration and angular velocity-based parameters (RMS and peaks) are in line with previous literature, even if a direct comparison can be hardly performed due to the differences between the considered motor tasks, experimental protocols and data processing techniques. As an example, Pratt and Sigward [65] quantified the shank angular velocity during single leg landing tasks and found peak values of about 200°/s on the sagittal plane. In the present study, peak values of the shank angular velocity magnitude ranged between 51 and 75°/s during a less impulsive task like the SLS.

In terms of reliability, parameters extracted from the acceleration trajectory on the tibial transverse plane (SP, SA, and SAecc) proved to be good candidates for a reliable and effective quantification of the knee stability during SLS. Similar considerations can be drawn for the root mean square values of both foot and leg angular velocity. Furthermore, the ICC values of those parameters were slightly lower that those reported by Alenezi et al. for both kinetic and kinematic parameters obtained through marker-based motion capture and force plates [66]. Interestingly, a remarkably low reliability was found for the RMS of the foot acceleration and the peak angular velocities of both foot and shank only in the non-dominant limb. Overall, a smaller variability was found for the dominant limb, for which ICC values ranged from 0.60 to 0.76. Differences in variability between dominant and non-dominant limbs have been widely documented within the existing literature during different motor tasks and on different parameters [67–69]. Our results confirm these findings and corroborate the hypothesis that higher variability may represent an attempt to improve compensation in motor control, specifically in multi-segmental tasks that require interjoint coordination [67]. Interestingly, this between-limb difference in variability is not mirrored by the LSI results, which ranged between 95 and 112%; thus, within a physiological range [58]. This result confirms the hypothesis that LSI of similar magnitude may be achieved by movement characterized by different variability [13] and suggests that merely reporting the LSI does not provide any information about the strategies used for generating the movement patterns [13,14,70].

In the CHT, the values of the covered distance were in line with previous studies considering young healthy athletes practicing open-skills sports (football, soccer, volleyball or basketball) [12] and showed high reliability [71]. When considering MIMU-based parameters, root mean square acceleration values (40 to 100 m/s^2^ during track running [72,73] Vs 62 to 82 m/s^2^ in the CHT landing) and peak vertical angular velocities at the shank (909°/s during jump landing tasks [26] Vs 704 to 830°/s during CHT landing) are in line with the existing literature.

When considering parameters reliability, the temporal parameters, with the exception of the flight time, and the angular velocity-based ones showed an overall greater reliability with respect to the acceleration-based counterparts. Again, even if less pronounced, greater ICC values were found for the dominant limb, with respect to the non-dominant one. It is worth underlining that higher reliability scores indicate that significant differences displayed by the considered parameter within the same participant can be ascribed to potential modifications of knee stability rather than to physiological intra-subject variability. This suggests that these parameters can be considered as good candidates for knee stability monitoring using the proposed instrumented protocol. Conversely, the low reliability may indicate that either the analysed components of the motor task are too variable for reasonable measurement, or the MIMU measurement error is not adequate for the specific parameter. Improving either of these options could improve the parameter reliability.

Finally, the computation of the MDC allows us to characterize the measurement error in order to provide the actual score of the methodology [74]. The found results are in line with the findings associated with the ICC and they should be used by practitioners in order to compare the changes in performance-based measure of function when assessing the knee stability. More specifically, the here reported MDC values must be always taken into account in case of follow-up measurements in order to understand if the revealed changes are effectively due to a worsening and/or improvement in the knee stability and not only to a random error associated with the measurement methodology.

The generalizability of the present study results must be interpreted in light of the following considerations: first, linear acceleration signals are highly influenced by impacts. On this line, prior studies examining running tasks have observed how different running shoes and surfaces might influence the values of the acceleration components [75–77]. However, in our study the tests were conducted always on the same surface and all the participants were instructed to wear standard sneakers, whose differences are assumed not to have significantly impacted the study outcome. Second, all the participants were non-professional male soccer players: further studies should focus on the application of the proposed protocol on athletes of different gender, level, and from other sports disciplines. Third, the relationship/correlation between some of the estimated parameters (typically the angular velocity-based ones) and indicators of ACL injury risk (such as the external knee abduction moment) was already reported in the literature [25,26,57]. However, this relationship should be further investigated and studies on a population of patients with ACLR is needed to strengthen and further corroborate the present findings. In order for the proposed protocol to be used in a real context, in fact, its capability to detect differences between healthy and ALCR athletes must be assessed. Last but not least, the squat depth was not standardized during the SLS test: this was done because forcing each participant to reach a predefined squat depth could potentially influence the way they performed the movement, which strongly depends on the subject flexibility and motor control [78].

## Conclusions

The present study focuses on the assessment of the reliability of sensor-based knee stability parameters during both single-leg closed-chain movements typical of open skill sports. This kind of information is indeed fundamental to set the basis for the implementation of a suitable and accurate testing procedure. The study shows the feasibility for the application of the proposed IMU-based protocol on a larger cohort of athletes with and without ACL reconstruction, with the aim of gathering information on the knee stability and on the likelihood to sustain an ACL injury (preventive role) or to incur in re-injury (when embedded in RTS test batteries). In this perspective, the proposed protocol represents a first step towards the definition of reliable and objective criteria for RTS clearance after ACL injury.
